# Lessons from the first U.S. school districts with real-time indoor environmental quality (IEQ) sensors in classrooms: benefits, barriers and opportunities

**DOI:** 10.1088/2752-5309/ae5fc3

**Published:** 2026-05-26

**Authors:** M Pilar Botana Martinez, M Patricia Fabian, Kyleigh Gunn, Katherine H Walsh, Madeleine K Scammell

**Affiliations:** 1 Department of Environmental Health, Boston University School of Public Health, Boston, MA, United States of America; 2 Boston Public Schools, Boston, MA, United States of America; 3 Institute for Global Sustainability, Boston University, Boston, MA, United States of America

**Keywords:** indoor environmental quality, indoor environmental monitoring, environmental health literacy, data literacy, schools, climate resilience, sustainability

## Abstract

With aging buildings, rising temperatures, and limited budgets, school districts in the United States need effective strategies to address classroom environmental issues, secure funding for upgrades, and develop climate resilience plans. Collecting real-time indoor environmental quality (IEQ) data in classrooms can support these goals, yet few districts have adopted comprehensive school environmental monitoring, and little is known about the barriers and facilitators to its use. Understanding the value of this approach to ensure good environmental conditions inside schools, the challenges faced by early adopters, and strategies to overcome them will be essential for broader implementation. During the spring of 2025, we interviewed 13 school staff members from 5 public school districts across the United States that were early adopters of IEQ monitoring. Discussions covered perceptions of IEQ in their districts, benefits of collecting this data, and barriers and facilitators to implementing sensors and leveraging the data collected. Participants identified many uses for classroom IEQ data, including supporting operations (e.g. simplifying environmental audits, remotely validating complaints, holding mechanical ventilation vendors accountable) and sustainability efforts (e.g. advocating for capital upgrades, monitoring wildfire smoke infiltration, predicting mold growth, ensuring medication safety, and student education). Opinions were mixed on publicly sharing the data. Barriers included database complexity, the need for environmental health (EH) and data expertise, high costs, technical challenges, workload, and ethical concerns. Schools addressed these challenges through vendor support, artificial intelligence, and partnerships with academic researchers. Key opportunities to advance environmental equity in schools include improving EH literacy among staff and leadership and establishing indoor air quality standards. This study highlights lessons learned from pioneering districts and demonstrates how IEQ monitoring can improve classroom conditions, support operations and funding, and guide climate resilience and sustainability initiatives. The findings offer valuable insights for school districts, governments, and researchers considering installing environmental monitoring in classrooms.

## 1. Introduction

Each day, fifty million children in the United States (U.S.) attend Kindergarten to 12th grade (K-12) public schools, spending approximately 6–10 h inside their classroom environments [1]. While classrooms that are free from pollutants and thermally comfortable are conducive to student well-being and optimal learning, poor indoor environmental conditions can negatively impact students’ health and disrupt learning [2–4]. The health impacts from these environmental stressors can result in school absences, compounding the negative effects on learning. While effective mechanical ventilation and thermal regulation systems can improve school classrooms’ air quality and keep temperatures comfortable, U.S. school buildings are aging [5, 6], and approximately 41% of school districts need to upgrade or replace their heating, ventilation, and air conditioning (HVAC) systems [7, 8]. Moreover, U.S. public schools face a $85 billion shortfall in funding, according to the 2016 State of our Schools Report [9, 10]. Against aging mechanical systems, funding shortages, and the absence of federal standards to regulate many of the environmental stressors in classrooms, school leaders are challenged to ensure good indoor environmental quality (IEQ) conditions for their students [1], and school staff members need tools to assess the environmental exposures in school classrooms.

During the COVID-19 pandemic, a few school districts worldwide installed IEQ sensors in their schools to identify classrooms that required enhanced ventilation to reduce viral transmission through measuring carbon dioxide (CO_2_) [11]. In addition to collecting CO_2_, many of these sensors measure other important environmental parameters such as carbon monoxide (CO), particulate matter (PM), volatile organic compounds (VOCs), temperature, and relative humidity (RH), offering the potential to assess overall environmental conditions in classrooms. Using IEQ data can help schools (a) flag unsafe peak levels of any parameters, (b) respond in real time to thermal issues in classrooms, (c) inform the need for mechanical systems adjustments and upgrades, or help evaluate interventions to improve environmental conditions [12]. For example, in a recent study, IEQ data collected in a large urban school district was used to identify classroom ventilation needs by characterizing inter- and intra-variability of CO_2_ in classrooms [13], and another study characterized CO_2_ in 11 schools in California, to evaluate approaches to improving classroom environmental conditions through HVAC retrofits [14]. CO_2_ data was also used in previous studies to estimate air exchange rates in thousands of classrooms [15], and temperature and RH data helped identify school cooling needs [16].

While these studies exemplify the potential value of collecting IEQ data in schools, this approach can pose challenges to implementation. District-wide continuous indoor monitoring collects large volumes of data requiring computing resources for data management and analytics, for which schools may not be ready. Ge *et al.* reported ∼250 million observations generated from district-wide data collection during an academic year, for only one environmental parameter [13]. Accordingly, the vast volume of data and its complexity has been identified in previous studies as a barrier to taking advantage of this strategic resource across organizations, compounded by the lack of data analytics skills among staff [17–19]. Lack of infrastructure to handle big data in schools, and limited IEQ data literacy among staff, may prevent schools from gaining valuable insights from the information collected.

While collecting IEQ data in schools could be a promising tool to improve classroom environmental conditions, adoption so far is low. A recent report investigating indoor air quality policies across the U.S. found that only three out of 23 large school districts in the U.S. had installed a considerable amount of environmental sensors in their buildings [20]. Low environmental health (EH) literacy could be another roadblock to taking advantage of IEQ data in schools. EH literacy has been defined by Finn and O’Fallon as ‘an emerging and evolving multidisciplinary field that seeks to better understand how individuals and communities make sense of and act on health-related information about environmental hazards,’ [21]. Poor EH literacy could be preventing school staff from taking actions to improve indoor environmental conditions inside schools, or taking advantage of the monitoring data if they are unable to interpret sensor readings to assess environmental exposures in classrooms. Previous research explored indoor EH and numeric literacy in residential settings [22, 23], but the topic of research deserves attention in schools, because acting on classroom IEQ data is entirely dependent on school staff members being able to understand the implications the environmental stressors have on the school occupants’ health and learning. Accordingly, a previous study explored teachers’ experiences with classroom ventilation in California K-12 classrooms, and identified gaps in knowledge about IEQ, which led to classroom occupants’ actions that negatively impacted air ventilation [24].

This study explores benefits, barriers, and opportunities experienced by school staff members working in U.S. school districts that were the first to install thousands of real-time IEQ monitors in their classrooms. We conducted interviews and extracted insights about their monitoring data initiatives, with a focus on gaps in EH and data literacy. Our findings provide guidelines to inform future comprehensive environmental monitoring initiatives for school districts worldwide.

## 2. Methods

We conducted one-on-one semi-structured interviews with school staff members working in districts across the U.S. where classroom indoor environmental data is currently collected, and used thematic analysis to identify benefits, barriers, and opportunities to using IEQ data.

**Recruitment:** We recruited participants using purposive and snowball sampling based on an initial list of contacts from staff working in schools or districts currently monitoring environmental parameters in their buildings. Names of potential participants were identified through existing contacts as well as from a landscape analysis of school districts in the U.S. (Gunn *et al* 2025) [20], and attendees of national and local convenings (e.g. Green Schools Conference, Greenbuild) focused on indoor air and environmental quality in schools. All participants were invited to participate via email, and we asked them to refer us to other potential participants.

**Eligibility:** School staff were eligible if their roles were related to indoor environmental quality in classrooms, such as doing work inside classrooms (e.g., teachers), performing tasks related to improvements to indoor environmental quality conditions (e.g., mechanical systems operations staff, environmental and sustainability coordinators), or being concerned with health issues related to classroom environmental quality in classrooms (e.g., nurses, wellness coordinators). All participants had to be 18 years of age or older, fluent in English or Spanish, and able to consent for themselves.

**Figure 1.**
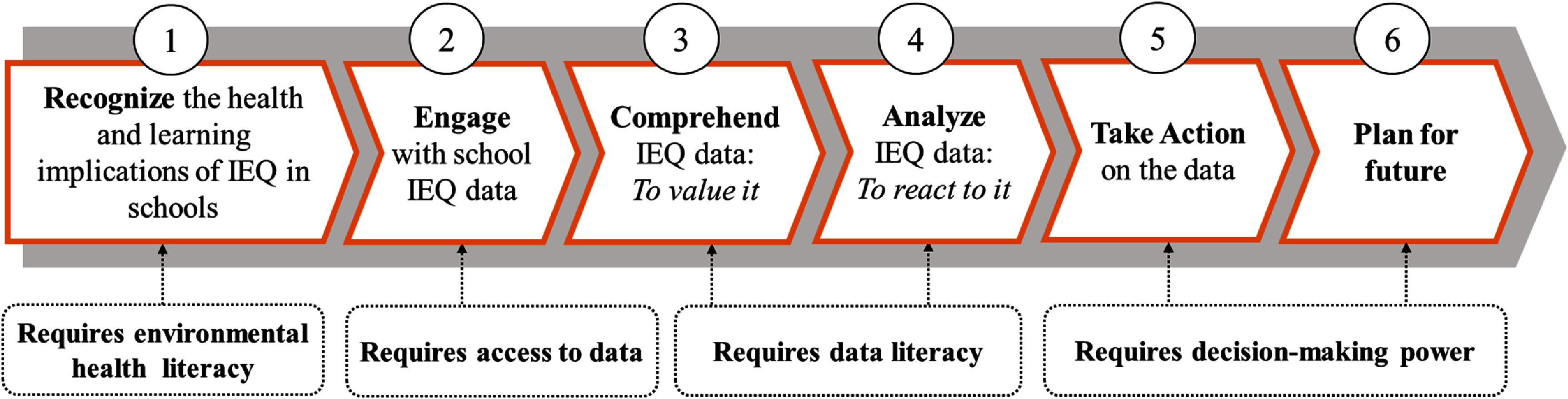
Environmental health and indoor environmental quality (IEQ) data literacy framework, adapted from Bloom’s taxonomy of educational objectives.

**Interview guide development:** The interview guide was developed based on a conceptual framework modeled after Bloom’s Taxonomy of Educational Objectives (1964) [25]. Our adapted framework delineates the steps from basic awareness of the implications of IEQ on health and learning, to the ability to act on the data for decision-making. See figure 1.

The interview was designed to ask general questions of all participants, as well as role-specific questions tailored to individual stakeholders. The interview was formulated with open-ended questions focused on pre-established domains [26]. The resulting interview guide had four key domains with various follow-up questions and probes (see supplemental materials). The interview domains were: (a) Perceptions and knowledge about how classroom environmental conditions impact school occupants, (b) Staff ability to influence IEQ in classrooms, (c) Access to school environmental data and perceived value of this information, and (d) Ability to interpret the IEQ data. Questions from the latter domain were only posed to participants who had access to the data and reported using it. The complete interview guide is included in the supplemental materials. Domains and questions for the interview were informed by a previous study on web content analysis to explore the state of environmental quality policies among U.S. public school districts [20], and notes taken from a workshop led by researchers in this study on the topic of leveraging IEQ data for operations, conducted during summer 2024 in one of the districts that participated in the study. We piloted the interview guide internally to evaluate clarity, flow, and time, per Chenail and Kallio *et al’s* recommendations [27, 28]. See a sample of questions in table 1.

**Table 1.** Sample questions for each of the four domains in the interview guide.

Interview domains	Interview questions
Perceptions and knowledge about how classrooms IEQ impact school occupants	• What do you think about the quality of the indoor air in classrooms in your school and district? • What factors, inside or outside, do you think may cause classrooms to have bad air quality? • How do you think that air quality impacts students or teachers in school classrooms, if at all? • What does ‘thermal comfort’ mean to you? What influences thermal comfort, in your opinion? • Do you think thermal comfort is important in school classrooms? Can you describe why or why not?

Staff ability to influence IEQ in classrooms	In your role, as a ___, how much can you influence the quality of the air? • What can you do personally? What do you have power to do? • What can you ask school staff to do? • Who do you ask to make a change? Or where you can look up information?

Access to school IEQ data and perceived value of this information	• Are you aware that your school/district has installed sensors to monitor in your school/district ? • What can you tell me about the data being collected? • Do you know if there is any place where you could look at the data being collected? The monitor display? A website?

Ability to interpret the IEQ data (These questions were only asked to those with access to the IEQ data)	• How would you describe your understanding or interpretation of the data shared with you? • Within your organization, do you know if there are trainings to learn how to interpret air quality and temperature data, for school staff? If so, for whom? • Is there a specific individual or team in your organization that exists to help you interpret these data?

***Interview protocol:*** Our study protocols were approved by the Boston University Medical Campus (BUMC) Institutional Review Board (IRB). At the beginning of the interview, participants verbally consented to participate. Interviews were conducted by research staff, in person or virtually through Zoom. Another researcher joined the interview to take notes when possible. With the participant’s permission, we audio-recorded and saved the interview, together with the notes, on a secure drive identified by the participant’s identification number. Participants did not receive any incentive.

***Qualitative data analysis:*** Interviews were transcribed by RevTranscription [29], an IRB-approved professional transcription service, reviewed by the interviewer to ensure transcription accuracy, and imported into NVIVO 15 (Version 15.1.1). The transcripts of the interviews were coded independently by two analysts based on a predefined codebook [26]. We used a combination of deductive and inductive reasoning to code the transcripts. The codebook was informed by the interview questions; however, it expanded during the coding process, adding new codes as new insights emerged until we reached code saturation (Table S.1). To improve inter-coder reliability, weekly meetings were held between analysts to establish consensus on the codebook. We moved from codes to insights through inductive reasoning by identifying patterns and relationships between data categories. During the coding and analysis phases, both analysts practiced ‘memoing’, the act of recording thoughts, reactions, and interpretations while coding, and collaborated in the crafting of the final findings and selection of exemplary quotes. The completed manuscript was sent to participants for feedback and results were compiled to share with each of the school districts included in this study.

We calculated inter-rater reliability at the end of the coding process with percentage agreement and Cohen’s kappa coefficients [26]. Analysts wrote personal reflexivity statements on the biases they bring to the research [30]. Inter-coder reliability measured after coding completion was 97.8% based on % coding agreement, and the Cohen’s kappa coefficient was 0.65, considered good in previous literature [31]. Lessons from the first U.S. school districts with real-time indoor environmental quality (IEQ) sensors in classrooms: benefits, barriers and opportunities.

## 3. Results

Interviews were conducted between March and May of 2025. Twenty people were invited to participate and 13 completed interviews. The participating public school districts included: Boston, Massachusetts; Denver, Colorado; Montgomery County, Maryland; Clark County, Nevada; and Prince William County, Virginia, which are among the first districts in the U.S. to have implemented comprehensive, continuous, live IEQ monitoring in classrooms. All are considerably larger than average school districts in the U.S. (which have ∼5.6 schools and <3000 students) [32], have centralized systems to collect environmental complaints and prioritize repairs, and have implemented continuous environmental monitoring in their classrooms at least one year prior to the interviews. The amount of monitoring coverage varied from ∼2000 classrooms, to 100% classroom coverage. All monitoring systems in these school districts recorded CO, CO_2_, PM, temperature, and RH, and 4 also collected VOCs. Two districts published their IEQ data on a public dashboard. Details about each of the school/district’s characteristics and monitoring approaches are shown in the table 2, informed from the interviews and from manual web-content searches [33–37].

**Table 2.** Characteristics of the schools/districts included in the study at the time the interviews were conducted.

School district	#school buildings/ #students	IEQ monitoring coverage	Parameters monitored						Publicly shared/ aggregation level
			CO	CO2	PM	VOCs	T	RH	
Boston	132 / ∼ 49 000	All classrooms and offices	√	√	√	*x*	√	√	Yes/ at the classroom level

Denver	180 / ∼90 000	Sensors in all schools, ∼10% school sensing coverage	√	√	√	√	√	√	No

Montgomery County	215/ ∼160 000	All classrooms and offices	√	√	√	√	√	√	Yes/ at the school level

Clark County	400 / 300 000	2000 smart thermostats	*x*	√	*x*	*x*	√	√	No
		∼ 100 sensors, moved around as needed	√	√	√	√	√	√	

Prince William County	100 / ∼90 000	15% of schools, 25% of the building space	√	√	√	√	√	√	No

CO = Carbon Monoxide, CO2 = Carbon Dioxide, PM = Particulate Matter, VOCs = Volatile Organic Compounds, *T* = temperature, RH = Relative Humidity.

Participant job roles included educators (‘Student Education’), staff in school health-related roles such as nurses or wellness program leaders (‘Student Health’), and staff in school operations roles. Within the operations group, there was a sub-group focused on energy efficiency and sustainability (‘Operations: Energy/Sustainability), and another dedicated to providing environmental services (‘Operations- Environmental Services’). Approximately half of the participants were from Boston Public Schools, half were operations staff. Participants were fairly evenly distributed across age categories, and the majority had college or higher degrees. Participants’ key characteristics are described in the table 3. Additional details can be found in Table S.2.

**Table 3.** Study participant characteristics (*n* = 13).

School district	Boston Denver Montgomery County Prince Williams County Clark County	54.8% (*n* = 7) 23.1% (*n* = 3) 7.7% (*n* = 1) 7.7% (*n* = 1) 7.7% (*n* = 1)

Job category	Student Health Student Education Operations: Energy/Sustainability Operations: Environmental Services	23.1% (*n* = 3) 15.4% (*n* = 2) 30.8% (*n* = 4) 30.8% (*n* = 4)

Participant age (years)	25–34 35–44 45–54 55–64 65–74	15.4% (*n* = 2) 15.4% (*n* = 2) 46.2% (*n* = 6) 15.4% (*n* = 2) 7.7% (*n* = 1)

Highest education	High School College Master	7.7% (*n* = 1) 53.8% (*n* = 7) 38.5% (*n* = 5)

The participants of the study shared their perceptions of indoor environmental conditions in classrooms in the districts where they work, their understanding of how IEQ impacts student wellbeing and learning, their access to the information, and the extent to which they use the data, if at all. Participants who use the environmental data shared with us shared their perceptions about the value of collecting this data, and their level of comfort and challenges faced in interpreting this information.

• **Participants’ perceptions of IEQ in their schools/ classrooms:** Overall, participants felt that IEQ in their school districts was acceptable, but highlighted variability in environmental conditions between buildings. Most of the thermal issues in classrooms raised by participants were about temperatures being uncomfortably hot, either from lack of AC, or from heating being too strong. Participants with Student Education roles expressed some level of frustration about the time it took for school facilities to address their complaints about IEQ issues in their rooms. Seven participants in non-education roles thought that some of the environmental issues in classrooms could be attributed to occupants’ behaviors (teachers and students), as discussed in the key findings. Participants also listed various air pollutants they experienced in their schools/ classrooms. Of these, some were generated from the buildings—such as mold (cited by 7), asbestos (cited by 6), dust from clutter (cited by 2), or furniture emissions (cited by 1). Other pollutants stemmed from classroom occupants’ practices and behaviors, such as perfumes or deodorants (mentioned by 6), air fresheners brought by school staff (cited by 4), vaping (cited by 3), cleaning products brought by classroom occupants (cited by 2), or whiteboard markers and art supplies (cited by 2). The most frequently shared outdoor sources were wildfires (cited by 4), and pollution from traffic or nearby factories (cited by 2).
• **Participants’ perceptions of how IEQ can impact school occupants:** Eight participants felt that poor environmental conditions in classrooms reduced learning. They mentioned reduced focus, attention, concentration, or creativity, as the mechanism of action. They also discussed the negative health implications of poor IEQ, including asthma (cited by 5), other respiratory conditions (cited by 1), or headaches (cited by 1). Three mentioned the compounding effect that absenteeism due to sickness has on learning. Participants also discussed how some school occupants may be more susceptible to poor indoor environmental conditions, such as those with asthma or neurodiverse students. Two participants mentioned the youngest students as being the most affected by heat, whereas another participant thought this population was more resilient to hot temperatures.
• **Education and sources of information about IEQ in schools:** Five participants (all with Operations roles) mentioned being aware of IEQ trainings/ educational sessions facilitated by the school district, such as webinars or certifications provided by third parties, and two of them specified that these trainings were voluntary, whereas the two staff members with Student Education roles were not aware of any trainings. Eight participants were able to cite sources of information about IEQ in schools. The Environmental Protection Agency (EPA) was the most cited source (mentioned by 5, all in Operations roles), while the EPA Tools for Schools, and U.S. Green Building Council (USGBC) were cited by 2. A participant in a Student Health role shared having provided internal training to other school staff members about the harms of poor environmental conditions for students in the past.
• **Ability to influence IEQ:** All participants in Operations roles reported having a moderate to strong ability to influence IEQ, whereas the Educators and those in student health roles, reported having limited or no control over IEQ. One staff in an Operations role explained how they had a great level of control over classroom environmental conditions in newer school buildings with HVAC systems that could be centrally managed and parameters could be adjusted by demand, while for older school buildings that relied on a boiler and radiators to temper the space, the only way to modify classroom environmental conditions was to ask occupants to open doors or windows and operate the window air conditioning (AC) units.
• **Level of engagement with the IEQ data:** All Staff members with Energy/Sustainability roles (*n* = 4) reported consulting the data systematically to inform their tasks, whereas only half of the participants with Environmental Services roles (2 out of 4) consulted the IEQ data frequently. Only one of the three staff members with Student Health roles consulted the data but only occasionally, and none of the participants with Student Education roles ever engaged with it.

Six key concepts surfaced from the analysis of the interviews: (1) innovative uses of the IEQ data, (2) limitations and solutions for schools to understand and act on the IEQ data, (3) opportunities to improve classroom environmental conditions by improving EH Literacy for school staff and leadership, (4) benefits and fears of sharing IEQ data publicly, (5) challenges to installing and operating environmental monitoring systems in a school district, (6) importance of equitable attention to aging school building systems and home environments.

**1. Innovative uses of IEQ data**

Participants in building operations roles agreed that IEQ data is useful to inform daily school operations. Several provided examples of how being able to consult the classroom data had improved the way they work. Specifically, three participants explained that having real-time data helps validate classroom occupant complaints without physically going to the location where the issue was reported. One participant mentioned how having monitors in classrooms simplified the environmental audit protocols by reducing the number of routine visits to classrooms and avoiding having to carry bulky instruments. For that reason, another participant in an Environmental role described having access to the IEQ data as *‘a game changer.’* Besides being used for daily operations, participants shared other potential uses for this data, such as monitoring the impact of a wildfire event inside classrooms or predicting mold growth before it is visible (2 mentions each). For others, the value of the data extended beyond identifying classroom IEQ issues. One staff member with a Sustainability and Energy role explained how data can help support decision-making and advocate for capital improvements, noting: ‘*you cannot dispute the data if presented properly.’*

One Energy and Sustainability staff member described using the data for detective work and vendor accountability. They were able to document that a recently purchased mechanical system was not designed correctly based on the IEQ data collected in the area served by the faulty unit. With evidence to support the claim, the school had no difficulty getting the unit replaced. Another participant described monitoring the temperature in the nurses’ offices without AC to ensure that temperature-sensitive medication stayed safe. Participants also proposed using the IEQ data as a source of education for kids (mentioned by 4). *It would be great to see this data used as part of the curriculum. Why not get teachers and students involved?* said a participant with a sustainability and energy role. Three participants had heard of similar educational initiatives leveraging other data sets, and one of them expanded on how data can be used to teach math and science, but also social studies, history, or even equity. As a staff member in a Student Health role put it, *‘What an amazing source of educational data for the kids, like real life. You can talk about equity, or you can talk about so many things with air quality data and with environmental data.’* Lastly, participants in Sustainability and Energy roles expressed interest in connecting the IEQ data with other datasets with the goal of improving school buildings’ sustainability operations. Three staff members discussed linking IEQ with school utilities, including electricity, natural gas (1 mention), and water (1 mention).

**2. Limitations and solutions for school districts to understand and act on IEQ data**

Everyone highlighted the value of continuous environmental monitoring in classrooms, and staff members who consult the IEQ data frequently (Operations roles) felt they had enough skills to explore the information. However, the size and complexity of the data was brought up by eight school staff members as a barrier to fully capitalize on this information, which requires EH and data science expertise. A staff member with a Sustainability/Energy role explained the limitations of making sense of the large volumes of data collected by the sensors. ‘*The data by itself is interesting, but does not tell a story.’* Lack of data literacy was pointed out as a barrier for the general public to use this information, *‘The challenge to these monitors and to the platform is that it takes a certain level of education and information to be able to interpret what you are reading,’* said one participant in a Student Health position. Participants who typically use the IEQ data reported that there was not a designated data science individual within their school district for managing and analyzing the classroom IEQ data (mentioned by 7). Specifically, a participant clarified that while there may be individuals in their organization with the necessary skills, analyzing IEQ data is not part of their current responsibilities, “*There are a couple of people who could do that. It is not their job. And that’s a distinction that I need to make.”* Three operations staff expressed the need for having such expertise in-house. However, currently, the responsibility for managing and analyzing the data is spread across different roles and layered over their current job responsibilities, limiting how much they can engage with the data.

When discussing how to act on the IEQ data, two participants lamented the fact that there are no federal school indoor standards for most of the environmental parameters measured with monitors. Consequently, school staff who use IEQ data in their job felt frustrated by the lack of decision-making power that this information offers when discussing building upgrades, comparing their experience in schools with when they previously worked as an environmental consultant, ‘*If I was cleaning up soil contamination or water contamination, I had a standard that I had to clean up the soil or groundwater to.’* This makes it much harder for staff to advocate for upgrades.

To address the data literacy gap, school districts are looking for help from outside. All reported receiving some support from the sensor provider companies. For some, the support consisted of an initial training to get familiarized with the monitors and system, and meetings to identify the need for service enhancements. For others, manufacturer support included a monthly data and analytics meeting and the development of algorithms to develop alerts, such as the prediction of mold growth. Two school districts secured help from academic partnerships to fill in the data science gap, and one participant expressed how helpful these partnerships were, while lamenting that the option of partnering with a university may not be possible for smaller school districts. Only staff members in the energy/environment roles engage with these outside partners (sensor providers and universities), and they described both types of collaborations as equally positive.

Given resource constraints and staff limitations, participants expressed uncertainty regarding a long-term solution to filling the data literacy gap. One staff member proposed creating a school position that focuses exclusively on handling and analyzing the data, while two participants suggested that the gap could be addressed by leveraging artificial intelligence (AI). Specifically, one participant discussed how school staff could take advantage of AI to summarize the classroom IEQ data, while the other one mentioned that AI capabilities were being explored by the sensor vendors directly, to develop tools to generate automated IEQ summaries, alerts, and queries.

**3. Opportunities to improve classroom environmental conditions by improving EH Literacy for school staff and leadership**

Participants noted that classroom occupants had a low understanding of how buildings work and how IEQ can impact student health and learning, which led to practices that negatively impacted classroom conditions. Student Health and Energy and Sustainability staff members shared examples of how classroom occupants’ behaviors were contributing to poor IEQ, such as cluttering rooms with stuffed animals, blocking air vents with decorations, or bringing in air fresheners, cleaners or personal products. One participant in an Energy/Sustainability role illustrated the issue of pollution from personal products in this quote:

“We can see all these VOCs increase as people come into the building in the morning. And then when you start occupying a classroom, they skyrocket. And then when people leave the classroom, they drop. And it’s a lot of personal care products and what we bring into the building.”

Classroom occupant behaviors were also mentioned by some as contributing to unnecessary energy expenditure at the detriment of schools’ budgets and reducing schools’ capacity to invest in upgrades. According to one participant in a Student Education role, classroom occupants turn on the AC or open windows in winter months to counteract the hot temperatures from the heating systems. The staff member described this situation as *‘a waste of energy.’*

Two participants reported that the school IEQ data had been misinterpreted at some point because of a lack of sufficient EH and data literacy, causing unnecessary worry among community members. Another participant expressed concern about potential misinterpretation consequences if data is made available publicly, with this quote: ‘*everybody is scared of things they do not know.’* (participant in an Energy/Sustainability role).

While all participants thought that providing EH education in schools would be beneficial, two people expressed concern that additional training could be a burden on staff. Specifically, a participant in a Student Health role wondered how this education could be effectively delivered to teachers or parents, without overwhelming them. Two participants in buildings operation roles regretted the lack of EH understanding among those with leadership roles and decision-making power. Accordingly, a participant with an environmental role suggested that EH trainings should start at the top, with heads of schools and principals.

**4. Benefits and fears about sharing IEQ data publicly**

We heard contrasting opinions about the value of sharing the data broadly. Three participants saw public sharing of the data as a way to build trust between school managers and the community. One of them—in a Student Health role—noted that during the COVID-19 pandemic, it had been important to offer transparency about air quality inside classrooms, but expressed concern that the complexity of the data could lead to misinterpretation. This participant also shared an experience where parents became alarmed after noticing daily spikes on the data dashboard, describing the difficulty of reassuring them that the school district has a team of people monitoring for environmental issues, and that short-term peaks in certain parameters are expected and not a cause for concern. ‘*I have had parents contacting me to ask: did you know that in our school at this day this was happening? What is the school doing about this?’*. A staff member with a Student Education role expressed indifference about the potential of consulting classroom IEQ data, a responsibility that should lie *with others: ‘I am not excited about checking that data to see if my classroom has clean air. I would rather have someone else inform me if the air was not safe’.* Releasing the IEQ data publicly was also seen as a source of conflict within members of the school community, and a risk to the school’s public image. One participant expressed concern that parents might become distressed upon learning that their child’s classroom had poorer air quality compared to others in the building, potentially leading to tensions between guardians and school leadership. A staff with a Student Health role also mentioned that some school leaders were hesitant to begin monitoring air quality due to concerns about the implications of the findings, ‘*So, some principals were like, I do not know if I really want an air quality sensor at my school. we do not know if we want to know this information because then it will look bad for us and people will not choose to come here.*’

**5. Challenges to implementing and operating IEQ systems in a school district**

*Ethical concerns*. A reason that prevents some schools from wanting to install IEQ monitors was the potential ethical concern from collecting environmental data for schools with limited resources if they discover environmental issues that cannot be remediated due to insufficient funding: ” *I know there’s always a concern from other school districts or other leadership, it is just like, well, we may not want to know because we cannot afford to fix it.”* shared a participant in a Student Health role.

*Technical issues*. Sensor malfunction, miscalibration, or loss of connectivity were reported by three Operations staff members as challenges to operating the IEQ system.

*High costs*. Other challenges preventing IEQ monitoring implementation were the upfront cost for the purchase and installation of the system, and maintenance costs associated with servicing. In fact, for some school districts, funding shortages were the reason for not having sensors in all classrooms (*n* = 2). Specifically, two staff members in Environmental Services roles lamented not having more monitors and highlighted the importance of having sensors in all classrooms. To work around this problem, school districts that own a limited number of sensors move the devices around to address different IEQ needs as they arise.

*Job overload*. Eight participants mentioned job overload, experienced by themselves or others in the community. Of these, three mentioned understaffed teams as a barrier to engaging with the IEQ data more often or deeply. One participant with a Sustainability/Energy role complained about the difficulty of catching all the environmental issues that arise, given the massiveness of the data combined with the small team, *‘With so much data, and there’s only three of us, we certainly do not catch everything. I think the bottleneck becomes how quickly you can review it.’*

**6. Importance of equitable attention to aging school building systems and home environments**

Most participants (*n* = 11) noted that there is a great variability in IEQ conditions between schools in their district, driven by differences in the age of buildings and aging mechanical systems, and emphasized how old the school buildings are in their district. ‘*The biggest concern we have is the age of the equipment that we are using in most of the buildings.’*, said a staff member in an Energy/Sustainability role. Similar statements were offered by participants in all of the school districts represented in this study. Those in Operations roles (*n* = 8) provided nuanced detail on the air quality issues related to old buildings and aging mechanical systems, such as HVAC units failing, asbestos from old construction materials, or infrequent air filter replacements. Most Operations staff pointed to a chronic lack of funding as the main reason for not being able to upgrade HVAC systems. One participant in an Environmental Services role shared feelings of frustration about the constant cycle of repairs,*‘[The mechanical systems] are not well maintained. There’s never any money to maintain them. They just break down. And so we are constantly just running out, putting Band-Aids on them.’* Understaffed teams, an overload of responsibilities, and reduced training were also cited by buildings operation staff as a barrier to keeping up with school repair demands, *‘not enough people, not enough training for the maintenance personnel, and then also not enough money, resources or budget allocated to things such as proper preventative maintenance.*’, said a participant with an energy/sustainability role. Four participants brought up the fact that there are schools in their district that do not have AC at all. According to a staff member in a Student Health role, the schools without AC in their district are having to close earlier on very hot days, as the only solution to cope with the lack of mechanical cooling, acknowledging how this contributes to disparities in learning by reducing students’ instruction time. Moreover, one participant lamented that nurses’ offices in these unconditioned buildings can reach temperatures too hot for kids to stay in safely for long periods.

Participants also identified inequities in the approaches to prioritizing school upgrades as noted by an Environmental Services staff member:

“ *The way that buildings get put on the list [to be fixed] or prioritized is people go to the board and the squeaky wheel gets the grease, not the school that maybe needs it the most, not the school where the students are suffering the most from poor ventilation.”*

**Figure 2.**
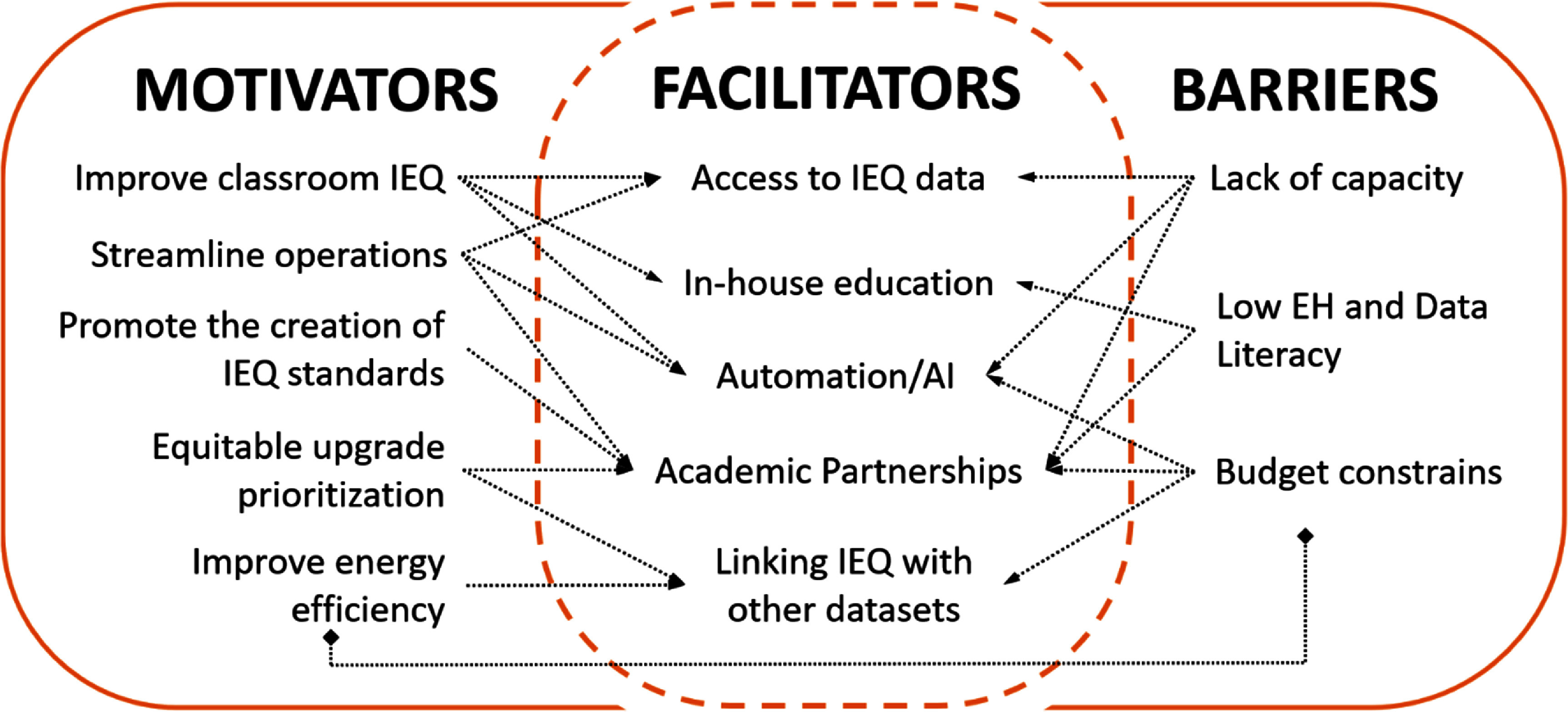
Motivations, barriers, and facilitators to leveraging classroom IEQ data for school staff.

Five participants shared their concern about inequities in the distribution of environmental exposures in the district. Two of them explained how schools with poorer IEQ conditions serve low-income families who live in neighborhoods that are overburdened by environmental stressors: ‘*it is a total equity issue. The older buildings that cannot afford AC are in the areas of town where the kids that are in lower socioeconomic status go to,’* said a participant with a Student Health role. Three participants were also concerned about the fact that for students living in heavily polluted neighborhoods, in sub-standard homes with no AC, environmental stressors do not end when they leave the school. Consequently, the tension between school staff’s desire to provide students with the best possible environments and the inequities in environmental exposures that some students face leads to frustration and despair (mentioned by 5), encapsulated in this statement, *‘This is the place where [the students] are supposed to be learning and getting an education, and we are not giving them the learning environment that they deserve.*’ figure 2 shows the key motivators, barriers, and facilitators to leveraging classroom IEQ data for school staff.

## 4. Discussion

In this study we interviewed school staff members from the first U.S. school districts that installed real-time IEQ sensors in thousands of classrooms. To our knowledge, this is the first study to document the benefits, challenges, and opportunities of a large-scale real-time environmental monitoring system in schools.

### 4.1. Perceptions of the value of monitoring IEQ in schools

Participants identified a wide range of uses for classroom IEQ data, including supporting operations (e.g. simplified environmental audits, validating complaints remotely, HVAC vendor accountability) and sustainability initiatives (e.g. meeting energy goals), advocating for capital improvements, monitoring wildfire smoke infiltration, predicting mold growth, keeping medication safe, and leveraging data for student education. Using classroom environmental data to improve school building energy efficiency emerged from the interviews as a key motivator, aligning with a school staff survey in which facilities managers emphasized the value of tracking energy consumption and utility costs to reduce operational expenses, which has been discussed in previous literature [8, 14]. Connecting IEQ data with energy and utility information can support efforts to lower energy use and redirect cost savings toward school improvements.

Opinions were divided on the value of sharing data publicly. Some participants did not think that sharing the data publicly was necessary, or even desired. However, other participants thought that providing access to IEQ data to all school members could foster innovative ways to use this information. Viewed through an equity lens, broad data sharing is important to ensure it addresses community concerns—a key principle of participatory action research and community-based participatory research [38]. One reason some participants were reluctant to share the data publicly was the risk that people would misinterpret it, causing unnecessary worry or damaging the school’s image. However, this could be addressed if community access to IEQ data is accompanied by education that equips audiences with the tools to understand and act on the information.

### 4.2. Limitations to installing and managing IEQ systems

High costs, technical expertise, job overload, and the ethical implications of evidencing environmental issues that schools may not have funding to address were identified as barriers to installing and maintaining an IEQ sensor network. The constrained budgets and staff shortages are in alignment with a survey of school facilities managers [8], and a 2021 report on the state of the U.S. schools [9]. Additionally, all school districts included in this study are large, with centralized building operations systems, for which installing IEQ monitoring networks has made building operations and environmental services more efficient. However, this gain may not incentivize smaller schools with less complex operations. Additionally, for schools with very limited resources, installing IEQ monitors in all classrooms could present an ethical, and potentially a public image and legal conflicts, if they discover environmental issues but cannot remediate them due to insufficient funding. Resource constraints were also cited as barriers due to the cost of sensors, installation, and maintenance. As such, comprehensive IEQ monitoring may not be a realistic option for smaller school districts. Nonetheless, lessons learned from early adopters can guide others considering this approach. For example, purchasing only a few sensors that are relocated across the district as needed was discussed by our participants, which could be a scaled-down solution for schools not yet committed to district-wide IEQ monitoring. Ge *et al.* offers suggestions for the minimum number of sensors that should be installed in a school based on the total number of classrooms, so that estimates are not biased for 99% of the time, and this could be used to inform a partial-coverage monitoring approach [13].

As reported by participants, limitations to using the data included the database size and complexity, requiring in-house EH and data science expertise, and the diffuse responsibility for who manages and analyzes the data, which school districts are overcoming with support from sensor vendor companies, exploration of AI, and school-academic research partnerships. Specifically, some participants mentioned relying on monitors vendors to address their data management and analysis needs long-term. However, there could be some limitations to this approach, such as vendor lock-in situations where a school district is over reliant on the sensor provider, which may prevent the organization from switching companies as technology and needs change [19, 39]. Moreover, vendors may lack the EH expertise needed to extract insights relevant to student health and learning.

### 4.3. Solutions to address data literacy and capacity

On addressing the data literacy gap, two participants suggested AI as a solution to data management and analysis, either leveraged directly by the school staff, or embedded in the sensor’s vendors’ services. However, this approach also poses some limitations. While no-code AI platforms may empower the lay person with a means to tackle simple data analytics tasks without coding skills, complex analysis such as group comparisons or modeling requires statistical understanding to avoid issues that would compromise the results [19, 39]. Most importantly, the validity of any insight derived from data analysis depends on the data being accurate and EH expertise to validate and interpret the results [19, 39]. Thus, vendors’ business models that offer AI-powered solutions should include approaches to demonstrate data accuracy and a built-in data cleaning pipeline. Lastly, these practices could raise concerns about school and student data privacy, which needs to be considered carefully. In conclusion, while AI could help automate data summarization and simplify analysis, its use should be guided by school district priorities and focus on students’ learning and health needs.

To address EH and data needs, two school districts in our study formed partnerships with academic research institutions. These collaborations can fill data science and EH literacy gaps, and accelerate research on the links between classroom IEQ and student health and learning, which is critical for advancing school indoor environmental policies [40]. Such partnerships may also reduce schools’ need for reliance on vendors by serving as third-party auditors to assess sensor data accuracy. However, sustaining school-academic research partnerships over time poses challenges due to differing goals. While academic researchers seek novel contributions to science, schools often require routine analytics that may not meet research novelty standards. And while monetary compensation from the school to the researchers could be a motivator for the latter to stay in the partnership, this presents ethical concerns and introduces a conflict of interest to research done with the school’s IEQ data.

It is also relevant to discuss the meaning of these school-academic research partnerships from the perspective of community-engaged research (CEnR). This approach is defined by the involvement of the community in the research work, which is desired, as it offers communities a certain level of control to guide the research process to fulfill their needs [38]. However, defining ‘community’ in the context of school districts is complex. While the school-academic partnerships could be considered CEnR, the research is guided by school staff members, which is not representative of the entire school community, even if the partnership may bring benefits to all. As these partnerships evolve, it is important that both parties invest in developing sustainable, long-lasting relationships based on trust and supported by mutual benefit.

### 4.4. Advancing IEQ in schools

One key opportunity identified to advance IEQ is improving EH literacy across the school community. Participants in this study demonstrated high EH literacy overall by citing various IEQ stressors that were present inside their schools and articulating the health and learning implications of poor IEQ in classrooms. Participants also acknowledged that some populations—such as young students, school occupants with asthma, or neurodiverse students—were more susceptible to poor environmental conditions, consistent with existing literature [41–44]. However, insights from Operations and Student Health staff revealed notable EH literacy gaps among teachers and students, particularly regarding building mechanical systems illiteracy, in alignment with previous research [24], and the negative effects of aerosol cleaners, air fresheners, and personal products on classroom IEQ. The findings also highlight the need to prioritize EH education for school leaders, who have decision-making power to act on the IEQ data. Prior studies have shown that providing individuals with personal exposure data, alongside timely and tailored EH education, can encourage behavior change to reduce environmental exposures, an approach that could be replicated in schools [45]. Participants also emphasized the need for data literacy skills to fully leverage IEQ data, suggesting that data management and analysis training should be offered to school staff. However, the recurring theme of ‘job overload’ highlights the importance of tailoring training format and delivery to fit staff roles, needs, and capacity to act on the information.

A second key opportunity to improve IEQ is setting indoor air standards. Some participants identified the lack of IEQ standards as a major challenge in regulating environmental conditions in schools, making it more difficult for staff to advocate for upgrades. In the absence of health-based recommendations, some schools have adopted environmental policies based on guidelines intended for different populations. For example, residential heat and cooling regulations for rental housing in Massachusetts were adopted by BPS as the reference temperature for schools [12, 46], and OSHA recommendations for office thermal comfort were used as the basis for a proposed bill addressing school heat exposure in Connecticut [47]. More recently, New York public school district passed a bill that requires schools to take action when classroom temperatures reach 82 **°**F (28** °**C), and demands rooms to be vacated above 88 **°**F (31** °**C) [48]. Classroom IEQ monitoring could help accelerate research on the impact of environmental conditions on student health and learning outcomes, which in turn could guide the development of appropriate standards [40].

Lastly, inequities in school building infrastructure and exposures at school and at home were called out as areas needing focus in order to improve health and learning for those most vulnerable. Participants noted that students attending schools with poorer IEQ often face exposure to pollutants and extreme weather at home and in their neighborhoods. These disparities align with previous findings. For example, Chakraborty *et al* found disproportionate exposure to ambient pollutants among Black and Hispanic students in Florida, and Cheeseman *et al* reported disparities in pollutant concentrations by race, ethnicity, and income nationwide [49, 50]. Participants identified building age as a key contributor to these disparities, consistent with recent statistics from the National Center for Education Statistics, which report that the average U.S. school building is approximately 50 years old, with over 25% built before 1950 [5, 6]. Accordingly, our findings provide evidence for applying an equity lens when prioritizing school upgrades.

### 4.5. Recommendations to make the best of IEQ data in schools

Based on our findings and discussion, we identified three actionable recommendations for leveraging IEQ data in schools with indoor monitoring networks, organized according to the study framework shown in the figure 1.

1. To help school staff recognize the health and learning impacts of classroom IEQ, engage effectively with the data, and foster innovative uses of this information, schools should grant access to IEQ information to different stakeholders, alongside EH and data literacy education. However, the education should be delivered in ways that do not add burden to staff. Education should begin with those who can act on the data and be integrated into their roles to avoid extra workload. Using IEQ data as teaching material can also support EH literacy among students.
2. To fully realize the benefits of IEQ data, schools can link this information with other relevant school datasets. Combining this data with student needs, and learning and health outcomes can inform equitable prioritization of school upgrades and support epidemiological research on the relationship between classroom IEQ and student learning. By integrating IEQ data with student health and socio-economic information, schools could develop a prioritization index that accounts not only for the urgency of building improvements but also for the specific needs of the students they serve. Schools with only a small number of sensors can still take advantage of this approach to advance environmental equity by installing them in schools most in need, generating evidence to advocate for building and system upgrades. If schools pair the IEQ data with energy expenditure data, they can explore ways to reduce utility costs and greenhouse gas emissions.
3. To enhance their capacity for data science and analysis, schools can invest in sustainable research partnerships. It is crucial to clearly define roles and benefits for all parties to maintain a sustainable, long-term collaboration. Lessons learned from these pioneering districts should be shared with other schools currently using or considering IEQ monitoring systems.

**Figure 3.**
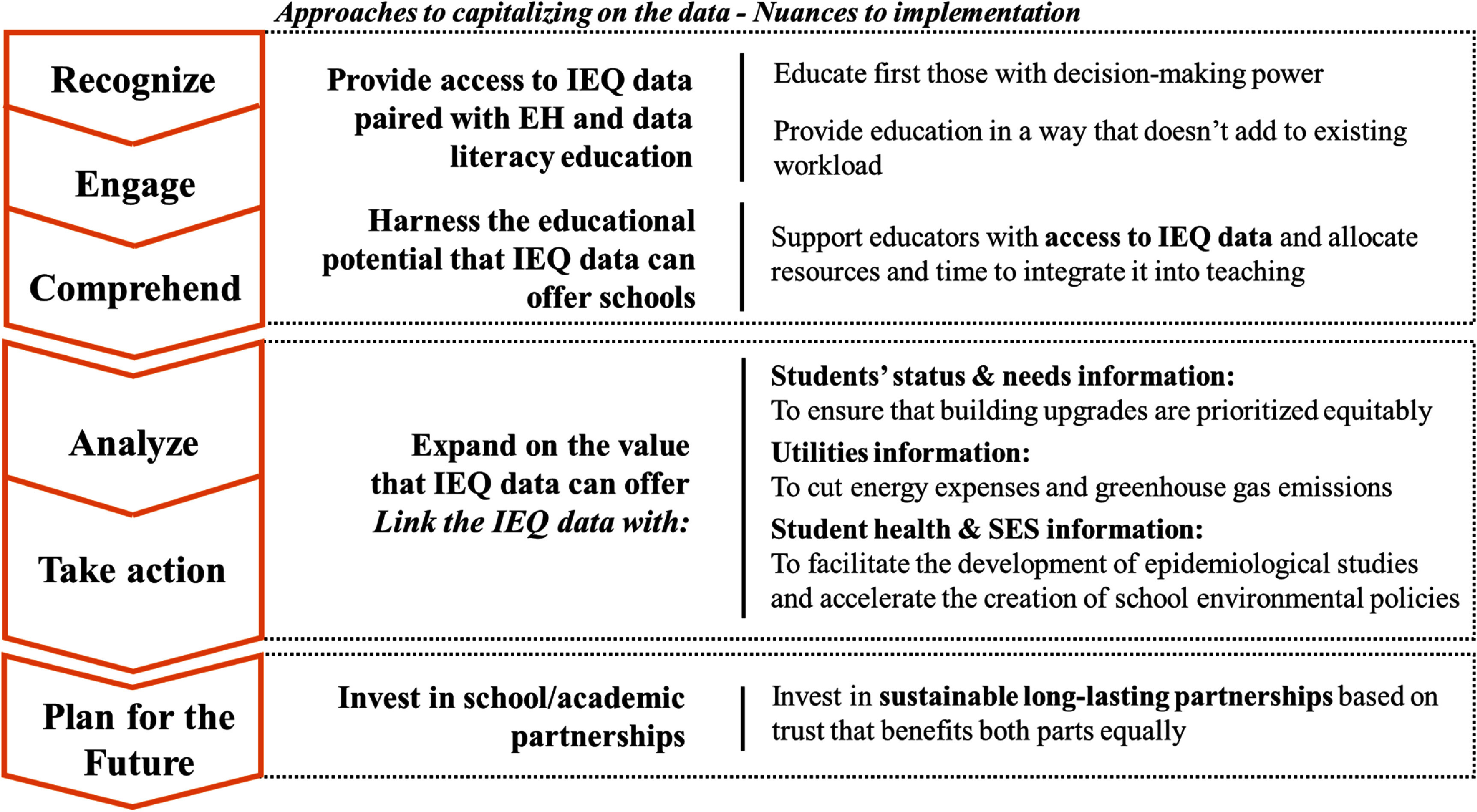
Approaches to capitalize on the IEQ data and nuances to implementation in relationship with the study framework.

These recommendations are summarized graphically, in relation to the framework of this study. Figure 3.

## 5. Limitations

This study explored barriers and facilitators to using classroom IEQ monitoring data, a novel and emerging practice. As a result, our school district sample was relatively small and unbalanced, with participants from districts of varying sizes and climates. Therefore, the findings may not be generalizable to all U.S. schools.

Using convenience and snowball sampling likely introduced self-selection bias, as those most interested in school IEQ monitoring may have been more motivated to participate and refer like-minded colleagues. The sample was also unbalanced in roles, with a majority of participants from Operations staff (*n* = 8)—the only group consulting IEQ data—limiting insights into barriers faced by educators or health staff. Most participants held college or master’s degrees, and over half were in management positions, which may not reflect the broader Operations workforce. Future research should further examine these barriers across different school staff roles, include more diverse and larger samples, and assess the specific data analytics needs of each group.

In order to increase the reliability of the study, the interviews were transcribed and cleaned based on audio recording. We had a second coder to enhance transparency and trustworthiness, who was less familiar with the project and had a different background to offer a fresh perspective on the data. The content analysis was guided by a codebook, and the two coders met regularly to review the codes and reconcile disagreements between them. Both coders practiced “memoing” and “reflexivity” during the coding and analysis process by documenting their thought process, and how their backgrounds, experiences, and opinions could be influencing their interpretation of the data. We measured inter-coder reliability based on 100% of the data coded. Despite the small and unbalanced sample, we found that similar themes emerged across different school districts, supporting the study’s validity. Overall, the goal of the study was not to aim for generalizability but to explore barriers and facilitators among early adopters.

## 6. Conclusion

This work elevates the knowledge of school staff from school districts that pioneered real-time IEQ monitoring and demonstrates the value that the comprehensive monitoring system can offer to school staff members, including improving classroom environmental conditions and students’ health and learning, supporting school building operations, improving energy efficiency, and informing climate resilience and sustainability initiatives. This work also highlights the importance of partnerships between schools and academic researchers. In partnership, these teams are able to turn the wealth of data collected by classroom monitors into valuable information for school districts worldwide. Gaining insight into the benefits of classroom environmental monitoring, the obstacles encountered by early adopters, and the approaches used to address them, is crucial for encouraging wider implementation. These findings can inform recommendations and best practices for school districts, governments and researchers that are considering IEQ monitoring in classrooms. Future study directions include scaling up the study with a large, balanced sample that includes all stakeholders in school IEQ, increasing sample sizes for students’ educators and those promoting student health, as well as capturing insights from students, parents and guardians, fomenting their involvement in the research through community engage research approaches. To the best of our knowledge this is the first study to explore barriers and facilitators to leveraging IEQ monitoring data in schools.

## Data Availability

The data cannot be made publicly available upon publication because they contain sensitive personal information. The data that support the findings of this study are available upon reasonable request from the authors. Supplementary information available at https://doi.org/10.1088/2752-5309/ae5fc3/data1.

## Data statement

The transcripts of the interviews contain information that could make a participant identifiable, and we didn’t get permission from participants to make their answers public; thus, we are not sharing the data for this study.
